# Beyond Chikungunya: Hidden risks of anti-mosquito chemicals

**DOI:** 10.1016/j.eehl.2026.100222

**Published:** 2026-02-11

**Authors:** Bin Hu

**Affiliations:** College of Environment and Climate, Institute of Mass Spectrometry and Atmospheric Environment, Jinan University, Guangzhou 510632, China

Since the summer of 2025, Chikungunya fever, a mosquito-borne infectious disease, has been ongoing in multiple Chinese cities such as Foshan, Guangzhou, and Shenzhen [[1], [2], [3]]. Given the current absence of effective methods to rapidly curb the spread of the disease, intensive insecticide use must be the dominant strategy for mosquito control [4,5]. However, the potential ecological and human health risks associated with large-scale and frequent use of insecticides have been overlooked in the pursuit of mosquito reduction.

Non-targeted and intensive use of insecticides presents multiple risks to ecosystems, e.g., accelerating the development of mosquito resistance, harming non-target organisms such as bees and butterflies, and adversely affecting other organisms in aquatic and soil environments [5,6]. These ecological risks could be further exacerbated in subtropical areas (e.g., Guangzhou) due to frequent rainfall events [3], especially when insecticide residues readily enter aquatic systems, creating pollutant dispersion beyond designated operation areas.

Furthermore, insecticides and their auxiliary materials are commonly applied as sprays or fumigants, resulting in high risks for human exposure through multiple pathways, including inhalation, ingestion, and skin contact, due to the contamination of air, water, food, clothes, and other media. More concerning is the fact that the long-term exposure to these insecticides may lead to toxicity effects and cumulative health consequences, including cancer and neurological issues [7].

To effectively control these risks, the timely detection of insecticide residues and their environmental impact is imperative. Currently, various portable state-of-the-art approaches can rapidly evaluate these risk factors by monitoring air, water, soil, and biological samples in field environments [8,9]. Furthermore, future mosquito control should adopt a one-two punch strategy by integrating sanitary urban management, new chemical-free technologies, and environmental remediation [3,4,10] to better control mosquito-borne diseases and enhance ecological and human health in a changing world.

## Declaration of competing interest

The author declares no competing interests.
